# Site-specific stabilization of minigastrin analogs against enzymatic degradation for enhanced cholecystokinin-2 receptor targeting: Erratum

**DOI:** 10.7150/thno.36746

**Published:** 2019-06-19

**Authors:** Maximilian Klingler, Clemens Decristoforo, Christine Rangger, Dominik Summer, Julie Foster, Jane K Sosabowski, Elisabeth von Guggenberg

**Affiliations:** 1 Department of Nuclear Medicine, Medical University of Innsbruck, Innsbruck, Austria; 2 Centre for Molecular Oncology, Barts Cancer Institute, Queen Mary University of London, London, United Kingdom

In our paper 1 unfortunately an error occurred in Figure 1. We therefore would like to provide the readers with the corrected figure and apologize for this inconvenience.

## Figures and Tables

**Figure 1:**
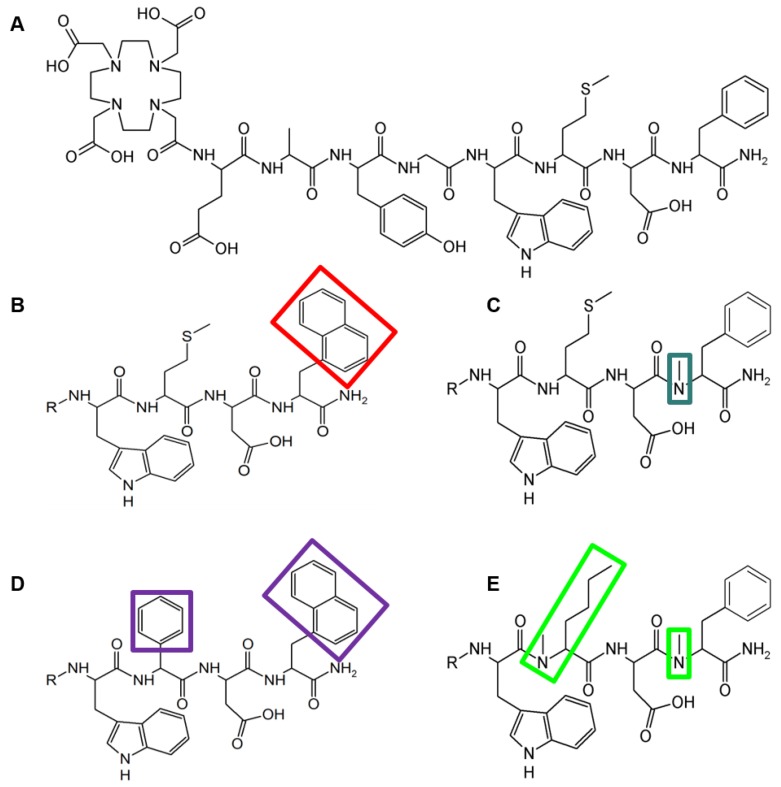
Structure of A) DOTA-MG11, B) DOTA-MGS1, C) DOTA-MGS2, D) DOTA-MGS3, E) DOTA-MGS4; R = DOTA-DGlu-Ala-Tyr-Gly.
